# Screening Prognosis-Related lncRNAs Based on WGCNA to Establish a New Risk Score for Predicting Prognosis in Patients with Hepatocellular Carcinoma

**DOI:** 10.1155/2021/5518908

**Published:** 2021-08-14

**Authors:** Xueliang Zhou, Mengmeng Dou, Zaoqu Liu, Dechao Jiao, Zhaonan Li, Jianjian Chen, Jing Li, Yuan Yao, Lifeng Li, Yahua Li, Xinwei Han

**Affiliations:** ^1^Department of Interventional Radiology, The First Affiliated Hospital of Zhengzhou University, Zhengzhou, China; ^2^Department of Neurology, The First Affiliated Hospital of Zhengzhou University, Zhengzhou, China; ^3^Department of Oncology, The First Affiliated Hospital of Zhengzhou University, Zhengzhou, China

## Abstract

**Background:**

Hepatocellular carcinoma (HCC) remains an important cause of cancer death. The molecular mechanism of hepatocarcinogenesis and prognostic factors of HCC have not been completely uncovered.

**Methods:**

In this study, we screened out differentially expressed lncRNAs (DE lncRNAs), miRNAs (DE miRNAs), and mRNAs (DE mRNAs) by comparing the gene expression of HCC and normal tissue in The Cancer Genome Atlas (TCGA) database. DE mRNAs were used to perform Gene Ontology (GO), Kyoto Encyclopedia of Genes and Genomes (KEGG) pathway analysis. Then, the miRNA and lncRNA/mRNA modules that were most closely related to the survival time of patients with HCC were screened to construct a competitive endogenous RNA (ceRNA) network by weighted gene coexpression network analysis (WGCNA). Moreover, univariable Cox regression and Kaplan-Meier curve analyses of DE lncRNAs and DE mRNAs were conducted. Finally, the lasso-penalized Cox regression analysis and nomogram model were used to establish a new risk scoring system and predict the prognosis of patients with liver cancer. The expression of survival-related DE lncRNAs was verified by qRT-PCR.

**Results:**

A total of 1896 DEmRNAs, 330 DElncRNAs, and 76 DEmiRNAs were identified in HCC and normal tissue samples. Then, the turquoise miRNA and turquoise lncRNA/mRNA modules that were most closely related to the survival time of patients with HCC were screened to construct a ceRNA network by WGCNA. In this ceRNA network, there were 566 lncRNA-miRNA-mRNA regulatory pairs, including 30 upregulated lncRNAs, 16 downregulated miRNAs, and 75 upregulated mRNAs. Moreover, we screened out 19 lncRNAs and 14 hub mRNAs related to prognosis from this ceRNA network by univariable Cox regression and Kaplan-Meier curve analyses. Finally, a new risk scoring system was established by selecting the optimal risk lncRNAs from the 19 prognosis-related lncRNAs through lasso-penalized Cox regression analysis. In addition, we established a nomogram model consisting of independent prognostic factors to predict the survival rate of HCC patients. Finally, the correlation between the risk score and immune cell infiltration and gene set enrichment analysis were determined.

**Conclusions:**

In conclusion, the results may provide potential biomarkers or therapeutic targets for HCC and the establishment of the new risk scoring system and nomogram model provides the new perspective for predicting the prognosis of HCC.

## 1. Background

In addition to being the sixth most common cancer in the world, liver cancer is also the fourth leading cause of cancer death, with 841080 new cases and 781631 deaths in 2018 [1]. In the United States, there were 42030 new cases of liver cancer and 31780 deaths in 2019 [2]. Due to the lack of special clinical manifestations in patients with early hepatocellular carcinoma (HCC), 70%–80% of patients are in advanced stages when they experience symptoms and have missed the opportunity for radical resection [3]. Although the current treatment of HCC includes surgical resection, transplantation, chemotherapy, radiotherapy, radiofrequency ablation, targeted therapy, transcatheter arterial chemoembolization (TACE), and immunotherapy, the overall survival rate has not changed significantly and the 5-year recurrence rate after surgery is still up to 70% [4]. Therefore, it is of great significance to clarify the molecular mechanisms of the occurrence and development of HCC and to identify new molecular markers to improve its clinical efficacy.

As a kind of noncoding RNA (ncRNA) without protein coding ability, long noncoding RNAs (lncRNAs) are transcripts more than 200 nucleotides in length [5]. Thousands of lncRNAs have been found due to the development of high-throughput RNA sequencing (RNA-seq). Originally, lncRNAs were thought to be the noise of genomic transcription, a byproduct of RNA polymerase II transcription, and have no biological function [6]. In fact, lncRNAs play important regulatory roles in different cellular processes, especially in different types of tumors [7–9]. Increasing evidence has shown that lncRNAs play a key role in the occurrence, invasion, and distant metastasis of HCC through cell differentiation regulation, epigenetic regulation, and cell cycle regulation [7, 10].

MicroRNAs (miRNAs) are a kind of noncoding RNA with a length of approximately 20–22 nucleotides. They can bind to a target protein coding gene in its 3′-untranslated region (3′-UTR) based on sequence complementarity, thus affecting the stability of the mRNA or interfering with protein translation [11, 12]. It was found that miRNAs are abnormally expressed and dysfunctional in a variety of tumors and play an important role in the occurrence and development of tumors, including gastric cancer [13], colorectal cancer [14], ovarian cancer [15], breast cancer [16], and HCC [17].

Weighted gene coexpression network analysis (WGCNA) is a systematic biological approach that describes intergene correlation patterns through RNA sequencing or microarray data [18]. It usually constructs a scale-free gene coexpression network to explore the correlation between the gene set and clinical characteristics, and it can recognize highly correlated genes and aggregate them into the same module [19]. However, many studies have only focused on the differential expression between genes, ignoring the high correlation between genes, and WGCNA can make up for these defects. Therefore, WGCNA plays an important role in identifying potential biomarkers or new therapeutic targets [20].

In recent years, studies have shown that the mutual regulation between lncRNAs and miRNAs plays an important role in the development of tumors [21]. The competitive endogenous RNA (ceRNA) hypothesis, first proposed in 2011, suggests that lncRNAs can act as ceRNAs to bind to miRNAs, affecting the regulation of miRNAs on target mRNAs and thus regulating the expression of related target genes [22]. As an open-source sequencing database, The Cancer Genome Atlas (TCGA) platform contains clinicopathological information and corresponding bioinformatics data for more than 30 types of human cancer, which is helpful for the comprehensive analysis of the regulatory function of the lncRNA-miRNA-mRNA ceRNA network in the pathogenesis of cancer [23]. These networks play an important role in understanding gene interactions and identifying potential biomarkers. However, the prognostic value of the lncRNA-related ceRNA regulatory network in HCC is still unclear.

In this study, we selected differentially expressed lncRNAs (DElncRNAs), miRNAs (DEmiRNAs), and mRNAs (DEmRNAs) between cancer and normal tissues by using data from the TCGA database. In addition, we also performed cluster analysis, biological function enrichment analysis, and pathway enrichment analysis on these DElncRNAs, DEmiRNAs, and DEmRNAs. Then, we constructed a coexpression network based on these differentially expressed genes (DEGs) to determine the modules related to clinical features by using the WGCNA-based systems biology method. According to the module data, the most relevant modules for survival and prognosis were selected to construct the ceRNA network. In this ceRNA network, there were 566 lncRNA-miRNA-mRNA regulatory pairs, including 30 upregulated lncRNAs, 16 downregulated miRNAs and 75 upregulated mRNAs. Moreover, we screened out 19 lncRNAs, and 14 hub mRNAs related to prognosis from this ceRNA network by univariable Cox regression and Kaplan-Meier curve analyses. Finally, we used the 19 prognosis-related lncRNAs to establish a new risk scoring system by lasso-penalized Cox regression analysis. The flow chart of the whole study was shown in supplement Figure S1. In addition, we established a nomogram model consisting of independent prognostic factors to predict the survival rate of HCC patients.

## 2. Methods

### 2.1. Data Collection and Preprocessing

HCC gene sequencing data were downloaded from the TCGA database (https://cancergenome.nih.gov/), and then, the original data were standardized for further analysis. First, the samples must have the detection data of lncRNAs, miRNAs, and mRNAs. Then, the samples that had other malignant tumors, no stage information (including pathological stage and TNM stage), and no age information were removed. Finally, 224 tumor samples and 27 normal samples were used for analysis. To identify DEGs between HCC samples and normal samples, such as lncRNAs, miRNAs, and mRNAs, we used a random variance model (RVM) to compare data between groups. The screening parameters of lncRNAs and mRNAs were set to *p* < 0.05, false discovery rate (FDR) < 0.05, and fold change (FC) > 1.5. The screening parameters of miRNAs were set to *p* < 0.05 and FC > 1.2. The data were obtained from the TCGA database and did not involve ethical issues.

### 2.2. Cluster Analysis of the DElncRNAs, DEmiRNAs, and DEmRNAs

The DElncRNAs, DEmiRNAs, and DEmRNAs were screened out, and cluster analysis was carried out according to the detected expression values in the samples, which were presented in the form of a cluster gram. The abscissa represents the sample, the ordinate represents the DEGs, red represents the high expression value of the DEGs in the sample, and blue represents the low expression value of the DEGs in the sample.

### 2.3. Functional Enrichment Analysis

The function of the DEmRNAs was analyzed using the Gene Ontology (GO) database (http://www.geneontology.org), and the signaling pathways involved in the DEGs were analyzed using the Kyoto Encyclopedia of Genes and Genomes (KEGG) database (http://www.kegg.jp/). Fisher's exact test and the multiple comparison test were used to calculate the significance level of each function and signaling pathway (*p* < 0.05).

### 2.4. Construction of the Weighted Gene Coexpression Network

According to the DElncRNAs, DEmiRNAs, and DEmRNAs and their expression information in HCC samples, cluster analysis was carried out to determine whether there were outlier samples. If they exist, the outlier samples need to be removed before analysis. WGCNA is a freely accessible R package [18] for performing weighted correlation network analysis. First, the optimal soft-thresholding power (*β*) value was calculated based on the expression data of the DElncRNAs, DEmiRNAs, and DEmRNAs in the HCC samples. Then, the coexpression matrix was constructed according to the soft threshold and the adjacency between genes was calculated. According to the similarity between genes, the coefficient of dissimilarity between genes was deduced and the cluster dendrogram of genes was obtained. Thus, the modules of lncRNAs/mRNAs and miRNAs were identified and the hierarchical cluster dendrogram of the genes in the module was displayed. Finally, according to the setting of the phenotypic information of the grouping traits, the correlation between the gene module and the phenotype was calculated and the trait-related module was identified.

### 2.5. miRNA Target Gene Prediction

The target gene mRNAs of miRNAs were predicted by miRanda (http://www.microrna.org/), TargetScan (http://www.targetscan.org/), and miRWalk (http://129.206.7.150/), and the corresponding target gene prediction results were obtained. The target gene lncRNAs of miRNAs were predicted by using miRanda and PITA (https://genie.weizmann.ac.il/pubs/mir07/mir07_exe.html), and the predicted results of the target lncRNAs that might be regulated by miRNAs were obtained.

### 2.6. Construction and Analysis of the ceRNA Network

According to the predicted miRNA-target binding relationship and the expression relationship in HCC samples, the negatively correlated miRNA-mRNA and miRNA-lncRNA pairs were screened. Combining the differentially expressed mRNAs, miRNAs, and lncRNAs, the coexpression network of the ceRNA (lncRNA-miRNA-mRNA) network was constructed. The lncRNA-miRNA-mRNA network was constructed and visualized with Cytoscape V3.7.

### 2.7. Protein-Protein Interaction (PPI) Network Analysis

The PPI network between the encoded proteins of the DEmRNAs was constructed by using the STRING protein interaction database. Protein interaction data from the STRING database were downloaded and imported into Cytoscape. Then, the protein interaction network was constructed using Cytoscape software.

### 2.8. Prognostic Gene Screening

HCC gene detection data with survival information were selected from the TCGA database. According to the expression of the DEGs in the sample, a univariate Cox regression model was used to analyze the relationship between the overall survival of HCC patients and the DEGs in the ceRNA network and *p* < 0.05 was considered to be significant. Then, the selected DEGs were visualized using the survival curve in the survival analysis.

### 2.9. Construction of the Risk Score System for Prognostic Prediction

After removing samples with missing survival time, 220 patients were randomly divided into two cohorts: a training cohort (*n* = 132) and a test cohort (*n* = 88). Then, univariate Cox proportional hazards regression was performed on lncRNAs in the training group and lncRNAs significantly related to OS in HCC patients were included in subsequent analysis. Next, lasso regression was used to select the potential risk genes and to eliminate genes that overfit the model. Finally, we used Cox proportional hazards regression to build a prognostic risk model. The calculation of the risk score used the following formula: riskscore = coefficient (gene1) × expression value of (gene1) + coefficient (gene2) × expression value of (gene2) + ⋯+coefficient (geneN) × expression value of (geneN). Using the median risk score of the training cohort as a cutoff value, all HCC patients were divided into a high-risk group and a low-risk group.

### 2.10. Establishment and Evaluation of the Nomogram Model for Predicting the Survival Rate of HCC Patients

Univariate and multivariate Cox regression analyses were used to screen independent factors related to prognosis, and a nomogram model was established and visualized for the obtained independent prognostic factors. To evaluate the prediction ability of the nomogram, we used resampling technology to carry out statistical inspection and drew the calibration curve. The closer the calibration curve is to the 45° line, the better the predictive power of the model constructed by the factors.

### 2.11. RNA Extraction and qRT-PCR

From January 2018 to January 2019, 10 pairs of liver cancer tissues and adjacent normal liver tissues were collected from 10 patients who underwent surgical resection and pathological confirmation in the First Affiliated Hospital of Zhengzhou University. The Medical Research Ethics Committee of the First Affiliated Hospital of Zhengzhou University approved this study. Total RNA was extracted by TRIzol reagent (TaKaRa), and cDNA was synthesized by reverse transcription using PrimeScript™ RT Master Mix (TaKaRa) according to the manufacturer's instructions. The levels of GAPDH, CTD-2510F5.4, and DSTNP2 were detected by qRT-PCR using SYBR@ Premix Ex TaqTM (Roche). The results were normalized to the expression of GAPDH. The primer sequences were synthesized by Servicebio (Wuhan, China). GAPDH-F: 5′-GGAAGCTTGTCATCAATGGAAATC-3′, GAPDH-R: 5′-TGATGACCCTTTTGGCTCCC-3′, CTD-2510F5.4-F: 5′-CACCATGCCTGGGTAATTTTAA-3′, CTD-2510F5.4-R: 5′-AGTTCCCTGTTGTCACTGACCTAT-3′, DSTNP2-F: 5′-TGGGCGAAGATGACCTGTTG-3′, DSTNP2-R: 5′-CTTGATTTCTTCTGGTGTGGAGC-3′. The relative expression levels of CTD-2510F5.4 and DSTNP2 were quantitatively calculated by the 2(−ΔΔ*CT*) method.

### 2.12. Statistical Analysis

All statistical analyses were performed using R software, and the Pearson correlation coefficient test was used to evaluate the rank correlation among the different variables. Kaplan-Meier curves and the log-rank test were used for survival data analysis. Univariate Cox regression analysis was used for survival factor analysis. Multivariate Cox regression analysis was used to determine independent prognostic factors, and a time-dependent receiver operating characteristic (ROC) curve was plotted to evaluate the accuracy of the prognostic prediction model.

## 3. Results

### 3.1. Clustering Analysis of DElncRNAs, DEmiRNAs, and DEmRNAs

**Figure 1:**
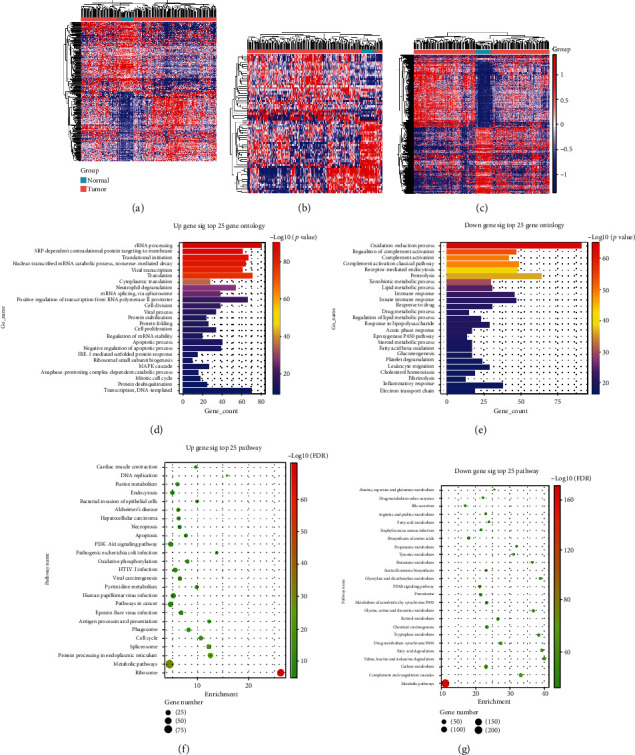
Heatmap for the hierarchical cluster analysis of (a) DElncRNA, (b) DEmiRNA, and (c) DEmRNA expression level changes between HCC samples and normal tissue samples. The rows represent DElncRNAs, DEmiRNAs, and DEmRNAs, whereas the columns represent the samples. Red represents the high expression of the DEGs, and blue represents the low expression of the DEGs in the sample. Top 25 enriched GO terms and KEGG pathways for DEmRNAs. (d, f) Upregulated genes; (e, g) downregulated genes.

According to the above screening criteria, 224 HCC samples and 27 normal samples were included. The screening parameters of lncRNAs and mRNAs were set to *p* < 0.05, FDR < 0.05, and FC > 1.5. The screening parameters of miRNAs were set to *p* < 0.05 and FC > 1.2. Finally, 1896 DEmRNAs, 330 DElncRNAs, and 76 DEmiRNAs (Supplemental Tables S1–S3) were obtained. According to the expression values of the DElncRNAs, DEmiRNAs, and DEmRNAs in the samples, cluster analysis was carried out and the results are displayed in the form of a heat map (Figures 1(a)–1(c)).

### 3.2. GO and Pathway Analysis of DEmRNAs in HCC

Compared with normal liver tissue, there were 1896 DEmRNAs in HCC tissues, including 953 upregulated DEmRNAs and 943 downregulated DEmRNAs. These upregulated and downregulated DEmRNAs were analyzed by using the GO database. These genes were enriched by terms in the GO database to determine their functions. The upregulated DEmRNAs were mainly enriched in rRNA processing, SRP-dependent cotranslational protein targeting to the membrane, translational initiation, nuclear-transcribed mRNA catabolic process, nonsense-mediated decay, viral transcription, and translation (Figure 1(d)), while the downregulated DEmRNAs were mainly enriched in the oxidation-reduction process, regulation of complement activation, complement activation, classical pathway, receptor-mediated endocytosis, and proteolysis (Figure 1(e)). Then, we performed KEGG pathway enrichment analysis and found that the upregulated DEmRNAs were mainly correlated with the ribosome, metabolic pathways, protein processing in the endoplasmic reticulum, spliceosome, and cell cycle (Figure 1(f)), while the downregulated DEmRNAs were mainly correlated with metabolic pathways, complement and coagulation cascades, carbon metabolism, valine, leucine and isoleucine degradation, and fatty acid degradation (Figure 1(g)).

### 3.3. Construction and Analysis of the Weighted Coexpression Network

**Figure 2:**
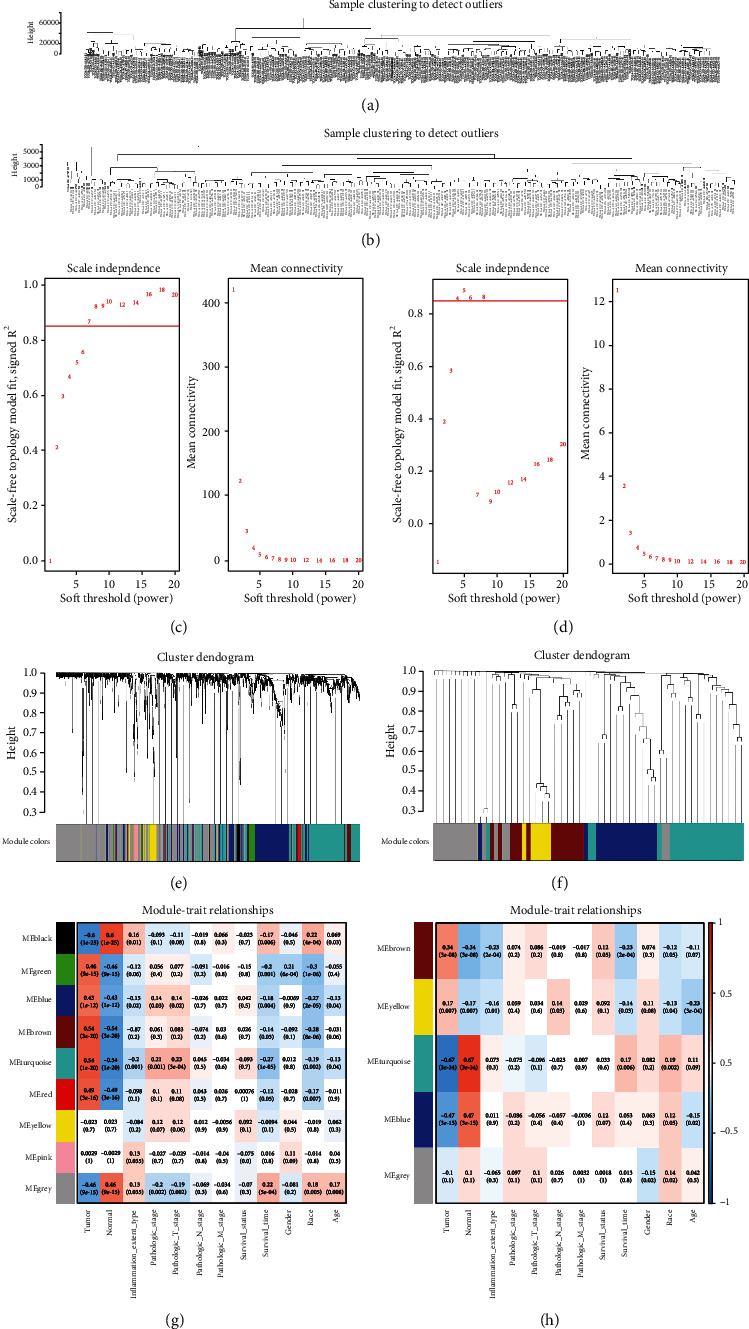
Sample clustering to detect outliers. (a) Represents lncRNAs/mRNAs; (b) represents miRNAs. Determination of the soft thresholding power in the (c) lncRNA/mRNA or (d) miRNA WGCNA. (c1, d1) Analysis of the scale-free fit index for various soft thresholding powers (*β*). (c2, d2) Analysis of the mean connectivity for various soft thresholding powers. Clustering dendrogram of (e) lncRNAs/mRNAs or (f) miRNAs based on a dissimilarity topological overlap matrix (TOM). Gray represents genes that are not classified into modules. Module trait relationships of (g) lncRNAs/mRNAs or (h) miRNAs were evaluated by correlations between module eigengenes and clinical traits. Each row corresponds to a consensus module, and each column corresponds to a trait. Each cell contains the corresponding correlation (first line) and *p* value (second line). The table is color coded (red: positively correlated; green: negatively correlated) by correlation according to the color legend. *p* < 0.05 represents statistical significance.

According to the expression information of the DElncRNAs, DEmiRNAs, and DEmRNAs in the samples, cluster analysis was carried out and the results showed that there were no outlier samples (Figures 2(a) and 2(b)). To determine the relative balance between scale independence and mean connectivity, the network topology with a soft threshold power of 1 to 20 was analyzed. Finally, we determined that the optimal *β* value of mRNAs/lncRNAs was 7 (Figure 2(c)) and the optimal *β* value of miRNAs was 4 (Figure 2(d)) in the coexpression network analysis. According to the consistent topological overlap and the corresponding module colors represented by the color row, a gene dendrogram was obtained by clustering the dissimilarity. Each colored row represents a color-coded module that contains a set of highly connected genes. Finally, nine modules were generated in the lncRNA/mRNA coexpression network (Figure 2(e)) and five modules were generated in the miRNA coexpression network (Figure 2(f)). Then, we calculated and mapped the relationship between each module and the corresponding clinical features. From the miRNA correlation module (Figure 2(h)), we found that the turquoise module had the largest negative correlation (module-trait weighted correlation = −0.67; the number of DEmiRNA = 22) with the tumors related to the miRNA coexpression network and had the largest positive correlation (module-trait weighted correlation = 0.17) with survival time. Therefore, for the miRNA module, we selected the turquoise module to construct the ceRNA network. In addition, from the lncRNA/mRNA correlation module (Figure 2(g)), we found that the turquoise module had the largest positive correlation (module-trait weighted correlation = 0.54; the number of DEmRNAs and the number of DElncRNAs are 481 and 48, respectively) with the tumor related to the lncRNA/mRNA coexpression network and the largest negative correlation with survival time (module-trait weighted correlation = −0.27). Therefore, in the lncRNA/mRNA module, the turquoise module was selected to construct the ceRNA network. Finally, the turquoise lncRNA/mRNA module was combined with the turquoise miRNA module to construct the ceRNA network.

### 3.4. Prediction of miRNA Target Genes and Construction of the ceRNA Network

**Figure 3:**
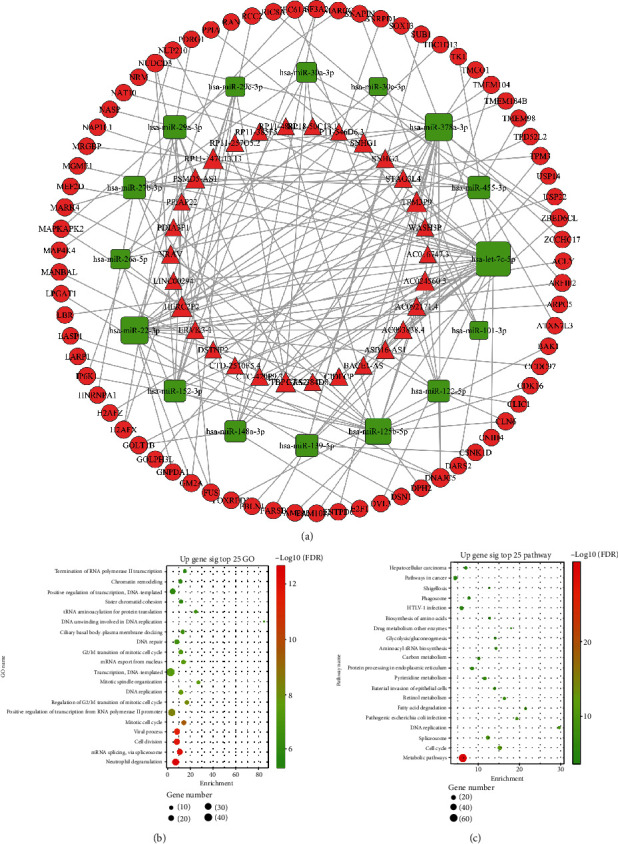
(a) The ceRNA network. The triangles represent lncRNAs, the rectangles represent microRNAs, the circles represent mRNAs, red represents the upregulation of DEGs in the tumor, and green represents the downregulation of DEGs in the tumor; the size of the shape represents the regulation ability of the gene, and a larger shape indicates a stronger regulatory capacity. The top 25 enriched (b) GO terms and (c) KEGG pathways for the 481 DEmRNAs in the turquoise module.

First, we performed target gene prediction for the miRNAs according to the above method. In the turquoise lncRNA/mRNA module and turquoise miRNA module, 196 mRNAs and 41 lncRNAs were predicted as the target genes of 33 miRNAs. Then, based on the predicted miRNA-target binding relationship and expression relationship in HCC samples, negatively correlated miRNA-mRNA and miRNA-lncRNA pairs were screened to construct the ceRNA (lncRNA-miRNA-mRNA) regulatory network. In the ceRNA network composed of the turquoise lncRNA/mRNA module and turquoise miRNA module (Figure 3(a)), there were 566 lncRNA-miRNA-mRNA regulatory pairs, including 30 upregulated lncRNAs, 16 downregulated miRNAs, and 75 upregulated mRNAs (Supplemental Table S4).

### 3.5. Functional Enrichment Analysis of the DEmRNAs in the Turquoise Module

The number of DEmRNAs in the turquoise module was 481. After module selection, we further performed functional enrichment analysis on the selected module. GO enrichment analysis showed that the 481 DEmRNAs in the ceRNA network (Figure 3(b)) were mainly enriched in neutrophil degranulation, mRNA splicing via the spliceosome, cell division, the viral process, and the mitotic cell cycle. KEGG pathway enrichment analysis showed that the 481 DEmRNAs in the ceRNA network (Figure 3(c)) were mainly correlated with metabolic pathways, the cell cycle, the spliceosome, DNA replication, and pathogenic *Escherichia coli* infection.

### 3.6. Construction of the PPI Network and Identification of 14 Prognosis-Related Hub Genes in the ceRNA Network

**Figure 4:**
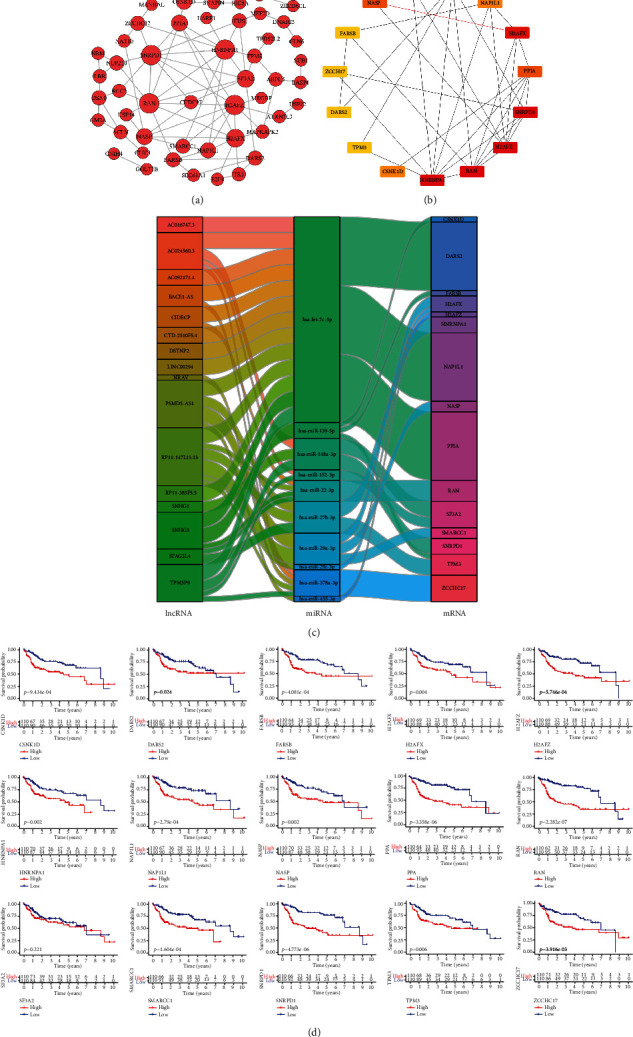
PPI network and Sankey diagram. (a) Red represents the upregulation of DEGs in the tumor, the size of the point represents the regulation ability of the gene, and a larger shape indicates a stronger regulatory capacity. (b) The top 15 hub genes. The darker the color of the hub gene is, the stronger the regulatory ability. (c) Sankey diagram of regulatory relationships among lncRNAs, miRNAs, and mRNAs. Left bar: lncRNAs; middle bar: miRNAs; right bar: mRNAs; DEGs: differentially expressed genes; lncRNA: long noncoding RNA; miRNA: microRNA; mRNA: messenger RNA. (d) Survival curves of the 15 hub genes associated with overall survival.

To further explore the relationship between the regulatory proteins of the ceRNA network, the PPI network was analyzed by the STRING database and the protein regulatory network was constructed by using Cytoscape software (Figure 4(a)). Finally, we selected the top 15 hub genes and constructed the regulatory network (Figure 4(b)). The ranking results of the top 15 hub genes are shown in Supplemental Table S5. Then, we visualized the relationship between the 15 hub genes and lncRNAs and miRNAs in the ceRNA network through a Sankey diagram. The results showed that the 15 hub genes were regulated by 16 prognosis-related lncRNAs (AC016747.3, AC024560.3, AC092171.4, BACE1-AS, CIDECP, CTD-2510F5.4, DSTNP2, LINC00294, PSMD5-AS1, RP11-147L13.13, RP11-385F5.5, SNHG3, TPM3P9, SNHG1, STAG3L4, and NRAV) through competitive binding with 10 miRNAs (hsa-let-7c-5p, hsa-miR-139-5p, hsa-miR-148a-3p, hsa-miR-152-3p, hsa-miR-22-3p, hsa-miR-27b-3p, hsa-miR-29a-3p, hsa-miR-29c-3p, hsa-miR-378a-3p, and hsa-miR-455-3p) (Figure 4(c)). Moreover, four (hsa-miR-139-5p, hsa-miR-148a-3p, hsa-miR-22-3p, and hsa-miR-29c-3p) of the 10 miRNAs were associated with good prognosis (Figure S2). To determine the relationship between these 15 hub genes and the prognosis of patients, Kaplan–Meier survival analysis and the log-rank test were used to evaluate the overall survival of HCC patients. The results showed that 14 (H2AFZ, HNRNPA1, RAN, SNRPD1, H2AFX, NASP, PPIA, CSNK1D, NAP1L1, DARS2, FARSB, SMARCC1, TPM3, and ZCCHC17) of the 15 DEmRNAs were considered to be important prognostic factors (Figure 4(d)) and the high expression of these genes was associated with poor prognosis in patients with HCC.

### 3.7. Screening of Prognosis-Related lncRNAs

**Figure 5:**
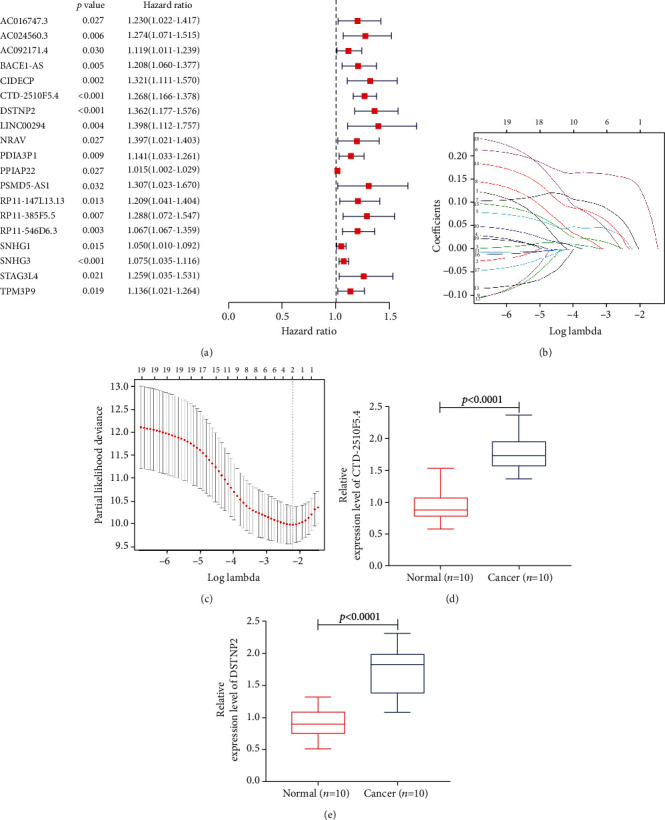
Prognosis-related lncRNAs and lasso regression. (a) Univariate Cox regression analysis screened 19 prognosis-related DElncRNAs. (b, c) Lasso-penalized Cox regression analysis of the 19 prognosis-related DElncRNAs. (d, e) The expression of CTD-2510F5.4 and DSTNP2 in liver cancer tissues and normal liver tissues was further verified by qRT-PCR.

Univariate Cox proportional hazards regression analysis was used to screen the prognosis-related lncRNAs in the ceRNA network. The results showed that 19 (AC016747.3, AC024560.3, AC092171.4, BACE1−AS, CIDECP, CTD−2510F5.4, DSTNP2, LINC00294, NRAV, PDIA3P1, PPIAP22, PSMD5−AS1, RP11−147L13.13, RP11−385F5.5, RP11−546D6.3, SNHG1, SNHG3, STAG3L4, and TPM3P9) of the 30 DElncRNAs were considered to be important prognostic factors (Figure 5(a)). Then, based on the 19 prognosis-related lncRNAs, we used lasso-penalized Cox regression and multivariate Cox regression analyses to select lncRNAs potentially related to prognosis and weighted their contribution according to the relative coefficient (Figures 5(b) and 5(c)). Finally, two optimal risk lncRNAs (CTD−2510F5.4 and DSTNP2) were selected and incorporated into the prognostic risk model. Furthermore, we further verified that the expression of CTD-2510F5.4 and DSTNP2 in liver cancer tissues was higher than that in normal liver tissues by qRT-RCR (Figures 5(d) and 5(e)).

### 3.8. Construction and Verification of the Prognostic Risk Model

**Figure 6:**
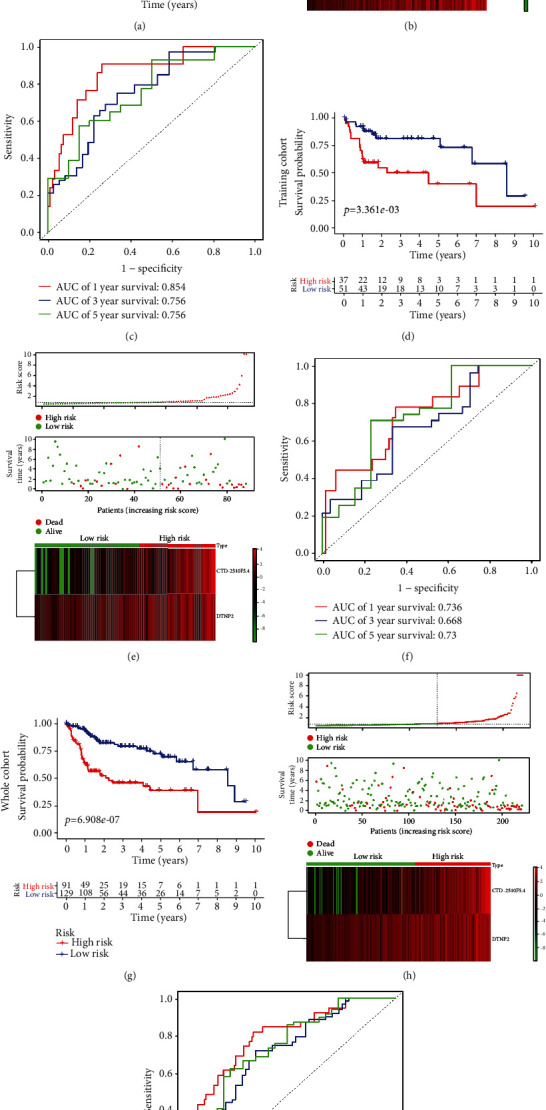
Prognostic analysis of the training cohort, testing cohort, and whole cohort. Kaplan-Meier curve analysis of the high-risk and low-risk groups in the (a) training, (d) testing, and (g) whole cohorts. Risk score distribution of the patients in the (b) training, (e) testing, and (h) whole cohorts. Time-dependent ROC curve analysis of the (c) training, (f) testing, and (i) whole cohorts.

To explore the significance of risk genes in predicting the prognosis of HCC patients, the risk score of each patient was calculated by the estimated regression coefficient and expression level of the risk lncRNAs. The calculation formula is as follows: training cohort risk score = (0.1895 × expression of CTD − 2510F5.4) + (0.1516 × expression of DSTNP2). According to the median risk score, patients in the training cohort were divided into a high-risk group and a low-risk group. We created a Kaplan-Meier curve based on the log-rank test, which showed that the prognosis of the high-risk group was worse than that of the low-risk group (*p* < 0.05) (Figure 6(a)). Then, we ranked the risk scores of the patients in the training cohort and generated a distribution map according to the survival status of each patient. A heat map was used to describe the expression of risk genes in the two prognosis groups (Figure 6(b)). Finally, we used a time-dependent ROC curve to test the accuracy of the model in predicting 1-, 3-, and 5-year overall survival. The area under curve (AUC) values of the prediction model were 0.854 at 1 year, 0.756 at 3 years, and 0.756 at 5 years (Figure 6(c)). To verify the accuracy of the prognostic risk model, we used it to analyze the test cohort and the whole TCGA cohort. Kaplan-Meier survival curve analysis in the test cohort (Figure 6(d)) and the whole TCGA cohort (Figure 6(g)) showed that the prognosis of the high-risk group was worse than that of the low-risk group (*p* < 0.05). The distribution of risk scores, survival status, and risk gene expression in the test cohort and the whole TCGA cohort are shown in Figures 6(e) and 6(h) and were similar to the results of the training cohort. The ROC analysis results showed that the AUCs at 1 year, 3 years, and 5 years in the test cohort were 0.736, 0.668, and 0.73 and the AUCs at 1 year, 3 years, and 5 years in the whole TCGA cohort were 0.798, 0.723, and 0.751, respectively (Figures 6(f) and 6(i)). These results indicate that our prognostic risk model can accurately predict the prognosis of patients with HCC.

### 3.9. Analysis of Independent Prognostic Factors

**Figure 7:**
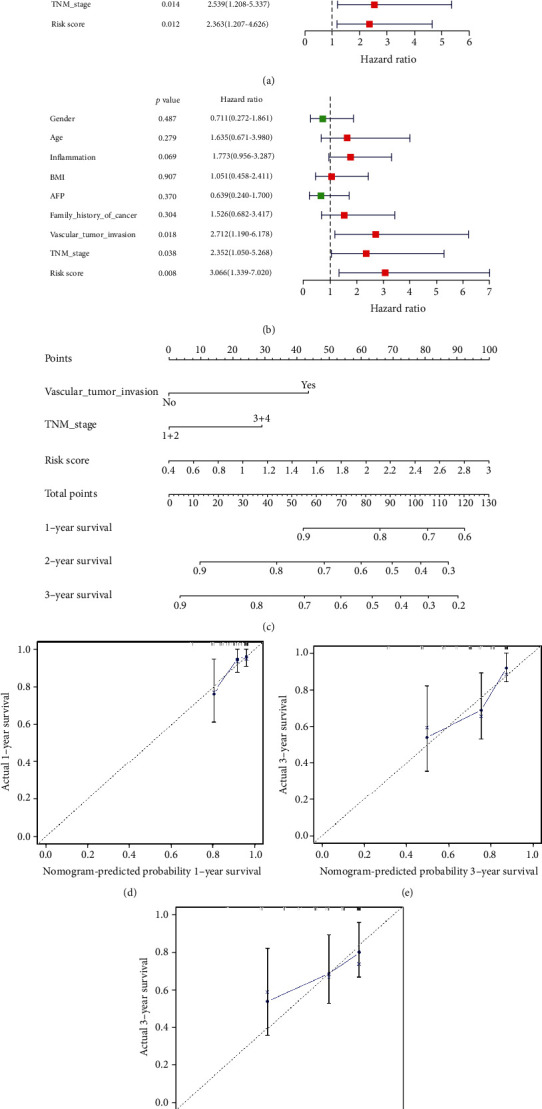
(a) Univariate Cox regression analysis and (b) multivariate Cox regression analysis for clinical factors. (c) Nomogram model consisting of the Vascular_tumor_invasion, TNM_stage and risk score factors for 1-, 3-, and 5-year OS prediction. (d–f) Calibration curves for the nomogram model of predicting 1-, 3-, and 5-year OS probabilities and the actual 1-, 3-, and 5-year OS probabilities.

Univariate Cox regression analysis showed that vascular tumor invasion, TNM stage, and risk score were related to the prognosis of patients with HCC (Figure 7(a)). To further confirm independent prognostic factors, we performed multivariate Cox regression analysis (Figure 7(b)). The results also showed that vascular tumor invasion, TNM stage, and risk score were significantly related to prognosis and might be considered independent prognostic factors.

### 3.10. Establishment and Evaluation of a Nomogram Model for Predicting the Survival Rate of HCC Patients

The three independent prognostic factors (vascular tumor invasion, TNM stage, and risk score) obtained above were further used to build a nomogram for predicting the 1-, 3- and 5-year survival rates of HCC patients (Figure 7(c)). From this nomogram, the 1-, 3-, and 5-year survival rates can be predicted according to vascular tumor invasion, TNM stage, and risk score. The calibration curve of the nomogram model showed that the predicted 1-, 3-, and 5-year overall survival rates were in good agreement with the actual survival rates, indicating that the nomogram model had good prediction ability (Figures 7(d)–7(f)). Moreover, we further evaluated the correlation between risk score and TNM and found that the higher the TNM staging, the higher the risk score (Figure S3).

### 3.11. Correlation between Risk Score and Immune Cell Infiltration, Gene Set Enrichment Analysis (GSEA), and 27 Immune Checkpoint Members

**Figure 8:**
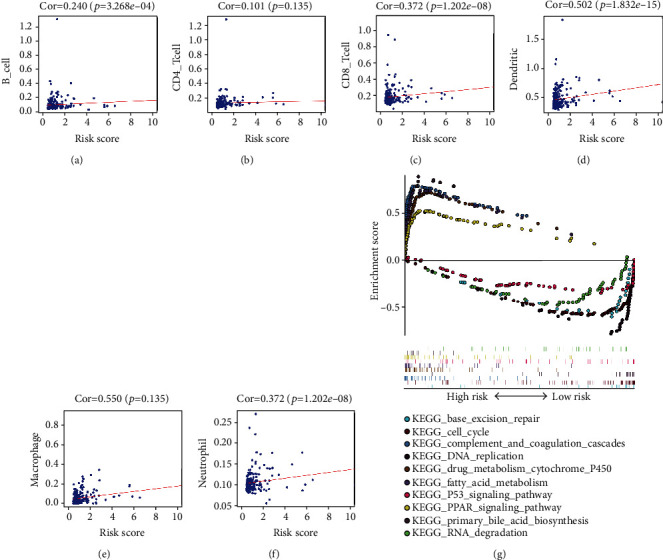
Analysis of the correlation between the risk score and immune cell infiltration in the entire TCGA cohort. (a) B cells. (b) CD4+ T cells. (c) CD8+ T cells. (d) Dendritic cells. (e) Macrophages. (f) Neutrophils. (g) Gene set enrichment analysis (GSEA) of the risk score in the TCGA dataset.

To explore whether our model can reflect the state of the tumor immune microenvironment, we analyzed the correlation between the risk score and immune cell infiltration in the whole TCGA cohort. The results showed that as the risk score increased, the content of immune cells (B cells, CD8+ T cells, dendritic cells, macrophages, and neutrophils) in HCC tissues also increased (*p* < 0.05) (Figures 8(a)–8(f)). Next, we explored the potential mechanism of the impact of the risk score on the prognosis of HCC patients through GSEA. The results show that the high-risk group is mainly enriched in complement and coagulation cascades, drug metabolism cytochrome P450, primary bile acid biosynthesis, fatty acid metabolism, and the PPAR signaling pathway; and the low-risk group is mainly enriched in DNA replication, base excision repair, the cell cycle, RNA degradation, and the P53 signaling pathway (Figure 8(g)). Finally, we evaluated the correlation between the risk score and 27 immune checkpoint members, including the B7-CD28 family (PD-L1, PD-L2, PD-1, CTLA4, CD276, HHLA2, ICOS, ICOSLG, TMIGD2, and VTCN1) [24], the TNF superfamily (BTLA, CD27, CD40, CD40LG, CD70, TNFRSF18, TNFRSF4, TNFRSF9, and TNFSF14) [25], and several other molecules (ENTPD1, FGL1, HAVCR2, IDO1, LAG3, NCR3, NT5E, and SIGLEC15) [26, 27]. The results showed that the risk score was positively correlated with the expression of PDCD1LG2 (PD-L2), CD274 (PD-L1), PDCD1 (PD-1), CTLA4, CD70, HAVCR2, CD276, LAG3, ICOS, ENTPD1, IDO1, HHLA2, CD27, TNFRSF9, TNFRSF18, and negatively correlated with the expression of FGL1 (Figure S4 and Supplemental Table S6).

## 4. Discussion

As the most common pathological type of liver cancer, the early symptoms of HCC are not obvious. Most patients are not diagnosed until they are in advanced stages; their prognosis is poor, and the treatment process can be a very painful experience. Therefore, it is very important to identify effective prognostic biomarkers and explore potential regulatory networks. The ceRNA hypothesis has been considered to be a new method of gene regulation through the competitive binding of miRNAs in HCC [28].

To further explore the regulatory network of prognosis-related molecules of HCC, we first used the TCGA database to identify the DElncRNAs, DEmiRNAs, and DEmRNAs in HCC and normal liver tissues. Then, GO and KEGG pathway enrichment analyses were carried out to further explore the main biological processes and regulatory pathways involved in these DEmRNAs. Next, we used WGCNA to identify the modules and selected the turquoise lncRNA/mRNA module and turquoise miRNA module, which were most closely related to the occurrence of HCC and the survival time of patients with HCC. Finally, based on the miRNA prediction website and the DElncRNAs, DEmiRNAs, and DEmRNAs in the above two modules, negatively correlated miRNA-lncRNA and miRNA-mRNA relationship pairs were constructed and a ceRNA network was generated by Cytoscape software. GO enrichment analysis revealed that the DEmRNAs in the ceRNA network were mainly related to mRNA splicing via the spliceosome, cell division, the viral process, and the mitotic cell cycle, and KEGG enrichment analysis showed that the DEmRNAs were mainly related to metabolic pathways, the cell cycle, the spliceosome, and DNA replication. Abnormal regulation of cell division and the mitotic cell cycle are key to the occurrence of cancer [29]. Moreover, many studies have shown that metabolic pathways play an important role in HCC [30, 31]. In this ceRNA network, we found that 30 lncRNAs may regulate the expression of 75 mRNAs by competitively binding 16 miRNAs. Then, the interaction among proteins was presented through the PPI network. Finally, the survival analysis of these 15 hub mRNAs showed that the high expression of 14 hub mRNAs (H2AFZ, HNRNPA1, RAN, SNRPD1, H2AFX, NASP, PPIA, CSNK1D, NAP1L1, DARS2, FARSB, SMARCC1, TPM3, and ZCCHC17) was related to the poor prognosis of patients with HCC. Before this, Bai et al. [32] found that PPIA may be a potential marker of gastric cancer, while Sun et al. [33] confirmed that the increased expression of DSN1 is related to the poor survival of patients with HCC. However, their mechanism of action has not been elucidated. Our study found that the regulatory axis of lncRNA SNHG3/mir-139-5p/DSN1 and lncRNA SNHG3/let-7c-5p/DSN1 may provide direction for the study of their mechanism of action. Moreover, some studies have shown that miRNA-2 promoted carcinogenic activity by upregulating the expression of RAN in HCC cells [34]; the high expression of HNRNPA1 promoted the invasion of gastric cancer cells [35]; lncRNA CDKN2B-AS1 promoted the growth of HCC by regulating the let-7c-5p/NAP1L1 axis [36]. Therefore, these results may also be the reason why the high expression of these hub genes is associated with poor prognosis in patients with HCC. Univariable Cox regression analysis of the DElncRNAs in the ceRNA network revealed that 19 lncRNAs (AC016747.3, AC024560.3, AC092171.4, BACE1−AS, CIDECP, CTD−2510F5.4, DSTNP2, LINC00294, NRAV, PDIA3P1, PPIAP22, PSMD5−AS1, RP11−147L13.13, RP11−385F5.5, RP11−546D6.3, SNHG1, SNHG3, STAG3L4, and TPM3P9) are related to the prognosis of patients with HCC. In recent years, the role of lncRNAs in a variety of cancers has been widely reported. Many experimental studies have shown that lncRNAs play an important role in many biological processes, such as cell cycle regulation, DNA damage, signal transduction, and epigenetic regulation [37]. Moreover, lncRNAs play a role through the competitive binding of miRNAs to regulate their target mRNAs [38]. For example, lncRNA SNHG3 can promote the progression of breast cancer [39], gastric cancer [40], lung cancer [41], laryngeal carcinoma [42], renal cell carcinoma [43], and liver cancer [44]. Zhang et al. [45] found that lncRNA SNHG3 induced EMT in HCC cells via miR128/CD151 cascade activation and that high expression of SNHG3 was associated with poor survival outcomes in HCC patients. Our research results also predicted that the high expression of lncRNA SNHG3 can be used as a biomarker of poor prognosis in patients with HCC and that SNHG3 may competitively bind multiple miRNAs to affect mRNA expression. Wang and Qin [46] found that lncRNA CTD-2510F5.4 may be involved in the pathogenesis of gastric cancer and has potential as a biomarker for the diagnosis and prognosis of gastric cancer. Similarly, we also found that the high expression of CTD-2510F5.4 was related to the poor prognosis of patients with HCC and has the potential to be a biomarker of HCC.

To identify the impact of these lncRNAs on survival prognosis, we constructed a risk prediction model. First, based on 19 prognosis-related lncRNAs, we used lasso-penalized Cox regression and multivariate Cox regression analyses to select two optimal risk lncRNAs (CTD−2510F5.4 and DSTNP2) and included them in the prognostic risk model. Then, the risk score of each patient was calculated by the estimated regression coefficient and expression level of the risk lncRNAs. According to the median risk score, the patients were divided into a high-risk group and a low-risk group. Kaplan-Meier curve analysis showed that the prognosis of the high-risk group was worse than that of the low-risk group. Miao et al. [47] also used lncRNAs to establish a risk model to predict the prognosis of elderly patients with non-small cell lung cancer. However, compared with their study, we used WGCNA to screen out the prognosis-related module and lasso-penalized Cox regression to further screen the optimal risk genes, which makes our risk model more reliable. Moreover, our model can reflect the state of the tumor immune microenvironment. With the increase in the risk score, the immune cells (B cells, CD8+ T cells, dendritic cells, macrophages, and neutrophils) in HCC tissue also increased. Finally, to better predict the prognosis of HCC patients at 1 year, 3 years, and 5 years after surgery, we constructed a nomogram based on the independent prognostic factors and the calibration curve showed that the nomogram model had good prediction ability. Of course, our research also has some shortcomings. First, we used data from a public database and the results still need to be further validated by prospective clinical trials. In addition, the mechanism of the prognosis-related DEGs affecting HCC progression needs further in vivo and in vitro experimental study.

## 5. Conclusions

In summary, we screened prognosis-related modules and constructed a ceRNA network through WGCNA. Then, we screened out 19 highly expressed DElncRNAs associated with poor prognosis in patients with HCC. Finally, a new risk scoring system was established by selecting the optimal risk lncRNAs from the 19 prognosis-related lncRNAs through lasso-penalized Cox regression analysis. In addition, we established a nomogram model consisting of independent prognostic factors to predict the survival rate of HCC patients. In conclusion, the results may provide potential biomarkers or therapeutic targets for HCC and the establishment of a new risk scoring system and nomogram model provides the new perspective for predicting the prognosis of HCC.

## Data Availability

All data generated or analyzed during this study are included either in this article or in the additional files.

## Abbreviations

HCC: Hepatocellular carcinoma
DEGs: Differentially expressed genes
DE lncRNAs: Differentially expressed lncRNAs
DE miRNAs: Differentially expressed miRNAs
DE mRNAs: Differentially expressed mRNAs
TCGA: The Cancer Genome Atlas
GO: Gene Ontology
KEGG: Kyoto encyclopedia of genes and genomes
ceRNA: Competitive endogenous RNA
WGCNA: Weighted gene coexpression network analysis
TACE: Transcatheter arterial chemoembolization
ncRNA: Noncoding RNA
RVM: Random variance model
FDR: False discovery rate
FC: Fold change
PPI: Protein-protein interaction
ROC: Receiver operating characteristic
AUC: Area under curve
GSEA: Gene set enrichment analysis.

## Supplementary Materials

Supplementary Materials Figure S1: the flow chart of the whole study. Figure S2: the 4 miRNAs were associated with good prognosis. Figure S3: the correlation between the risk score and TNM. Figure S4: the correlation between the risk score and 27 immune checkpoint members. Supplemental Table S1: differentially expressed lncRNA. Supplemental Table S2: differentially expressed miRNA. Supplemental Table S3: differentially expressed mRNA. Supplemental Table S4: 566 lncRNA-miRNA-mRNA regulatory pairs in the ceRNA network. Supplemental Table S5: the ranking results of the top 15 hub genes. Supplemental Table S6: the correlation between risk score and 27 immune checkpoint members.
