# Supplementary figures and images for: Screening Prognosis-Related lncRNAs Based on WGCNA to Establish a New Risk Score for Predicting Prognosis in Patients with Hepatocellular Carcinoma
Source: J Immunol Res. 2021 Aug 14;2021:5518908. doi: 10.1155/2021/5518908 (PMC8380184; doi:10.1155/2021/5518908)

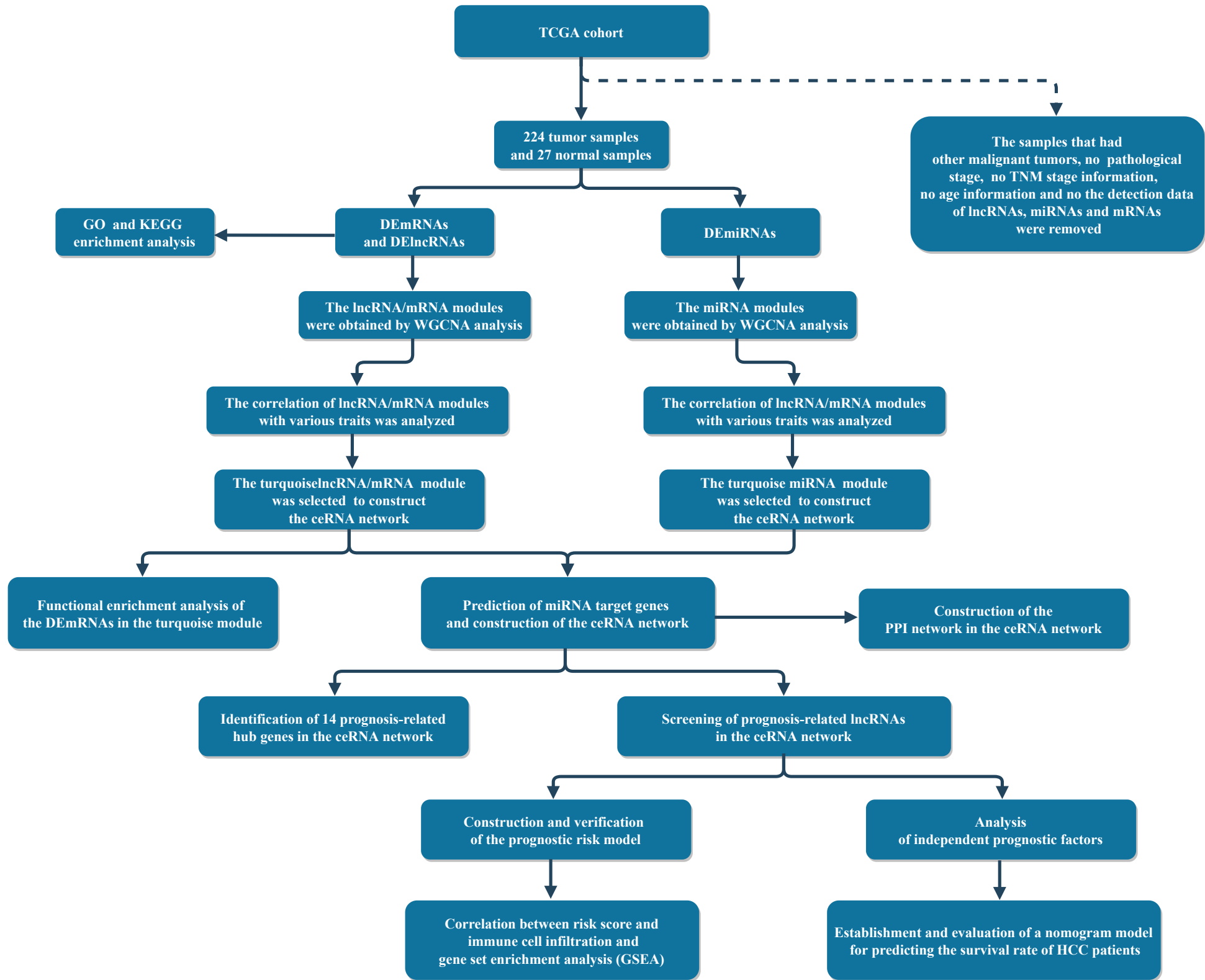

Supplement: Supplementary Materials — Figure S1: the flow chart of the whole study. Figure S2: the 4 miRNAs were associated with good prognosis. Figure S3: the correlation between the risk score and TNM. Figure S4: the correlation between the risk score and 27 immune checkpoint members. Supplemental Table S1: differentially expressed lncRNA. Supplemental Table S2: differentially expressed miRNA. Supplemental Table S3: differentially expressed mRNA. Supplemental Table S4: 566 lncRNA-miRNA-mRNA regulatory pairs in the ceRNA network. Supplemental Table S5: the ranking results of the top 15 hub genes. Supplemental Table S6: the correlation between risk score and 27 immune checkpoint members. [file 5518908.f1.zip › 5518908.f1/Figure S1 The flow chart of the whole study.pdf]

**hsa-miR-139-5p, p=0**

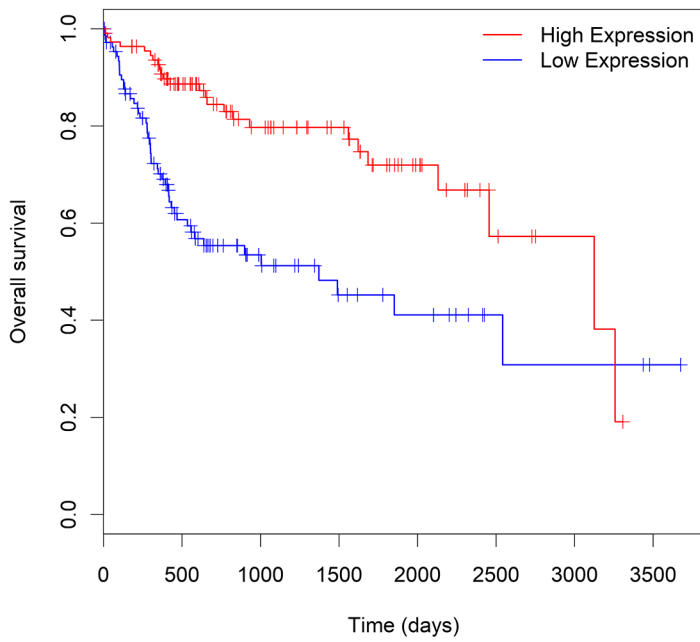

**hsa-miR-148a-3p, p=0.0464**

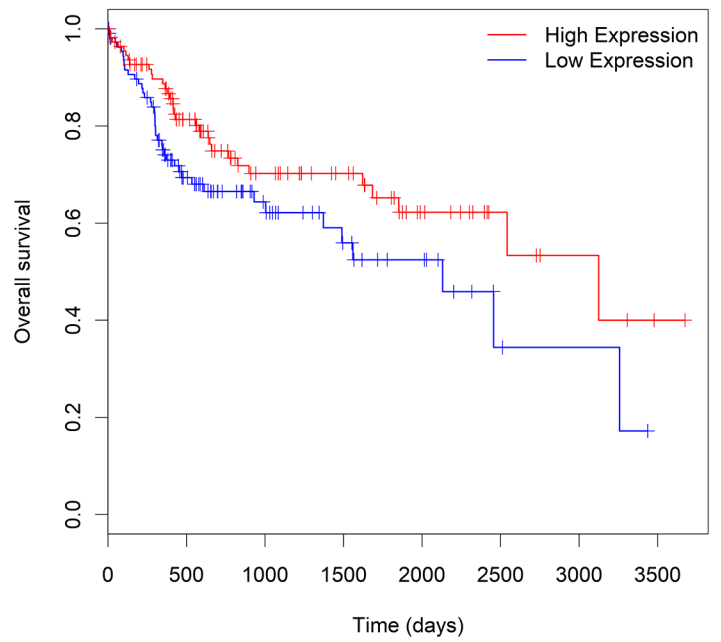

**hsa-miR-22-3p, p=0**

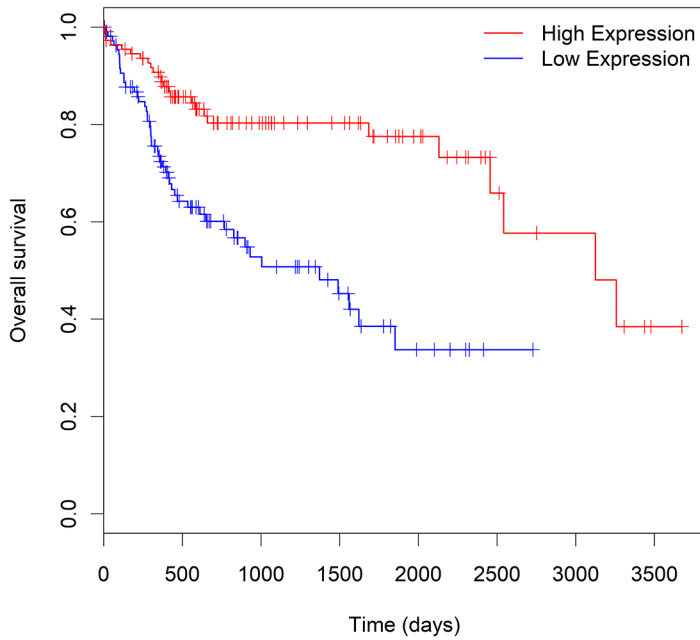

**hsa-miR-29c-3p, p=0.0305**

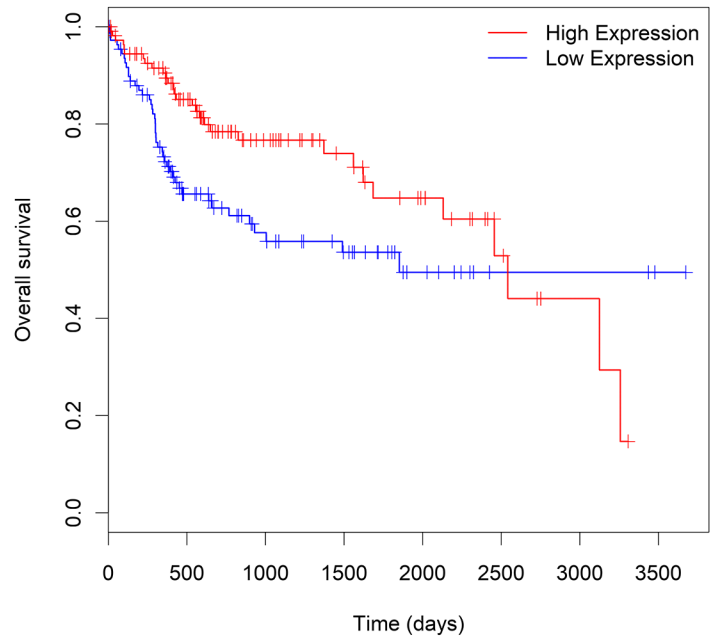

Supplement: Supplementary Materials — Figure S1: the flow chart of the whole study. Figure S2: the 4 miRNAs were associated with good prognosis. Figure S3: the correlation between the risk score and TNM. Figure S4: the correlation between the risk score and 27 immune checkpoint members. Supplemental Table S1: differentially expressed lncRNA. Supplemental Table S2: differentially expressed miRNA. Supplemental Table S3: differentially expressed mRNA. Supplemental Table S4: 566 lncRNA-miRNA-mRNA regulatory pairs in the ceRNA network. Supplemental Table S5: the ranking results of the top 15 hub genes. Supplemental Table S6: the correlation between risk score and 27 immune checkpoint members. [file 5518908.f1.zip › 5518908.f1/Figure S2 The 4 miRNAs were associated with good prognosis.pdf]

Anova,  $p = 2.3e-07$

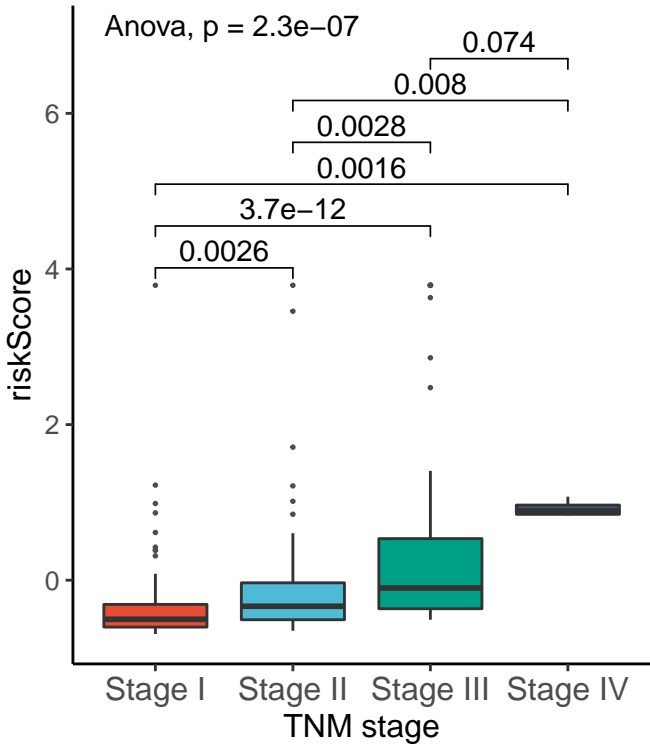

Supplement: Supplementary Materials — Figure S1: the flow chart of the whole study. Figure S2: the 4 miRNAs were associated with good prognosis. Figure S3: the correlation between the risk score and TNM. Figure S4: the correlation between the risk score and 27 immune checkpoint members. Supplemental Table S1: differentially expressed lncRNA. Supplemental Table S2: differentially expressed miRNA. Supplemental Table S3: differentially expressed mRNA. Supplemental Table S4: 566 lncRNA-miRNA-mRNA regulatory pairs in the ceRNA network. Supplemental Table S5: the ranking results of the top 15 hub genes. Supplemental Table S6: the correlation between risk score and 27 immune checkpoint members. [file 5518908.f1.zip › 5518908.f1/Figure S3 The correlation between risk score and TNM.pdf]

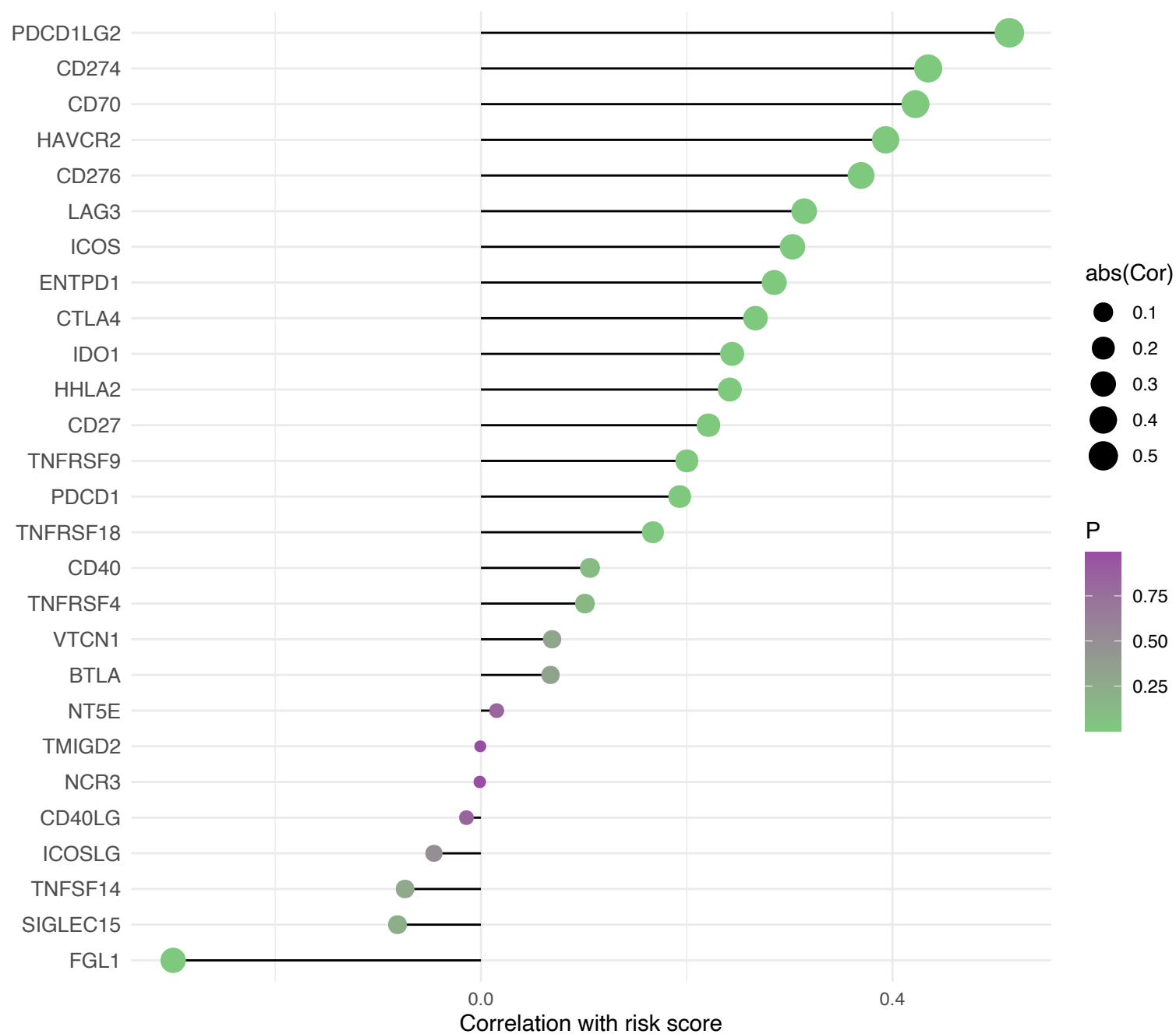

Supplement: Supplementary Materials — Figure S1: the flow chart of the whole study. Figure S2: the 4 miRNAs were associated with good prognosis. Figure S3: the correlation between the risk score and TNM. Figure S4: the correlation between the risk score and 27 immune checkpoint members. Supplemental Table S1: differentially expressed lncRNA. Supplemental Table S2: differentially expressed miRNA. Supplemental Table S3: differentially expressed mRNA. Supplemental Table S4: 566 lncRNA-miRNA-mRNA regulatory pairs in the ceRNA network. Supplemental Table S5: the ranking results of the top 15 hub genes. Supplemental Table S6: the correlation between risk score and 27 immune checkpoint members. [file 5518908.f1.zip › 5518908.f1/Figure S4 The correlation between risk score and 27 immune checkpoint members.pdf]
